# The Novel Application of Three-Dimensional Printing Assisted Patient-Specific Instrument Osteotomy Guide in the Precise Osteotomy of Adult Talipes Equinovarus

**DOI:** 10.1155/2021/1004849

**Published:** 2021-12-02

**Authors:** Yuan-Wei Zhang, Mu-Rong You, Xiao-Xiang Zhang, Xing-Liang Yu, Liang Zhang, Liang Deng, Zhe Wang, Xie-Ping Dong

**Affiliations:** Department of Orthopedics, Jiangxi Provincial People's Hospital Affiliated to Nanchang University, Nanchang, Jiangxi 330006, China

## Abstract

**Objective:**

This current research is aimed at assessing clinical efficacy and prognosis of three-dimensional (3D) printing assisted patient-specific instrument (PSI) osteotomy guide in precise osteotomy of adult talipes equinovarus (ATE).

**Methods:**

We included a total of 27 patients of ATE malformation (including 12 males and 15 females) from June 2014 to June 2018 in the current research. The patients were divided into the routine group (*n* = 12) and 3D printing group (*n* = 15) based on different operative methods. The parameters, including the operative time, intraoperative blood loss, complications, time to obtain bony fusion, functional outcomes based on American Orthopedic Foot and Ankle Society (AOFAS), and International Congenital Clubfoot Study group (ICFSG) scoring systems between the two groups were observed and recorded regularly.

**Results:**

The 3D printing group exhibits superiorities in shorter operative time, less intraoperative blood loss, higher rate of excellent, and good outcomes presented by ICFSG score at last follow-up (*P* < 0.001, *P* < 0.001, *P* = 0.019) than the routine group. However, there was no significant difference exhibited in the AOFAS score at the last follow-up and total rate of complications between the two groups (*P* = 0.136, *P* = 0.291).

**Conclusion:**

Operation assisted by 3D printing PSI osteotomy guide for correcting the ATE malformation is novel and feasible, which might be an effective method to polish up the precise osteotomy of ATE malformation and enhance the clinical efficacy.

## 1. Introduction

Talipes equinovarus (TE) is a kind of complicated whole lower limb deformity, which is mainly manifested as the plantar flexion and posterior talipes varus deformities, and in severe cases, it is often combined with anterior talipes adduction and arch elevation [1, 2]. The etiology of TE is often divided into primary and secondary, including the factor of specific gene deletion for the primary TE and the factors of congenital musculoskeletal malformations, neuromuscular diseases, trauma, infection, and burns for secondary TE [3, 4]. Meanwhile, the pathological changes of adult talipes equinovarus (ATE) malformation mainly include severe soft tissue contractures and bone and joint deformities [5, 6]. Hence, the overall complicated causes and abnormal deformity manifestations make the treatment require the comprehensive consideration of multiple factors. Currently, the nonsurgical treatment of TE has been generally recognized, and the Ponseti therapy has been widely applied in children with TE [7]. However, there is still no unified standard for the treatment strategy of ATE malformation, and the routine surgical methods mainly include Achilles tendon lengthening and balance muscle force of internal and external inversion, and the triple arthrodesis (TA) is also one of the most ideal surgical methods that is worthy of consideration [8, 9].

Since the TA was first proposed and applied in 1923 [10], it has gradually become one of the most important surgical methods for the correction of ATE malformation. However, ATE being a complex deformity, some of the patients after TA may be left with inferior outcomes, persistent deformities, and a variable degree of recurrence [11, 12]. Recently, with the in-depth understanding of ATE malformation, more and more scholars believe that the ATE malformation is a kind of three-dimensional and multidirectional anatomical relationship disorder [13, 14]. However, the pathogenic factors of ATE malformation are complicated, and the degree of deformities is not uniform. Moreover, the ideal treatment strategy for ATE malformation has not yet reached a consensus currently, which has presented a great challenge for surgeons to a certain extent [15]. During the operation, it is hard for operators to accurately adjust the osteotomy direction and angle of each dimension, so it often needs to repeatedly adjust or determine the correction strategy only based on subjective assumptions, which eventually leads to a huge deviation from the preoperative planning, and the prognosis will be unoptimistic. Hence, it is vital to develop an appropriate and personalized treatment plan for different etiology, deformity location, and degree of ATE malformation for the excellent functional reconstruction [16, 17]. With regard to this, in view of the unique advantages of three-dimensional (3D) printing in the personalized design and flexible application, it has been widely used in several orthopedic subspecialties, such as spine, joint, trauma, bone tumor, and orthopedics [18, 19]. Specifically, for one thing, 3D printing is particularly suitable for individualized customization and rapid manufacturing, which can greatly meet the special needs of orthopedic doctors for the implants. For another thing, 3D printing can also accurately and conveniently assist the routine operations in orthopedic surgeries, such as the osteotomy, reduction, and fixation, which improve the efficiency and quality of surgery to a certain extent [18, 20]. Thus, in order to further explore the novel surgical approach and evaluate the clinical efficacy of 3D printing assisted PSI osteotomy guide in accurate osteotomy of ATE malformation, this current research compared the routine ATE deformity correction surgeries with the operation assisted by PSI osteotomy guide and further evaluated the prognosis.

## 2. Methods

### 2.1. Patients

This retrospective research included a total of 27 patients (12 males and 15 females) of ATE malformation admitted to Jiangxi Provincial People's Hospital Affiliated to Nanchang University from June 2014 to June 2018. Therein, the inclusion criteria were mainly summarized as the patients diagnosed as the TE by preoperative physical examination and imaging examinations such as X-ray, computed tomography (CT), and magnetic resonance imaging (MRI). The exclusion criteria included (1) patients under 16 years old, whose bone development of the lower limbs had not yet been finalized; (2) the ulcer reached the bone level and formed the chronic osteomyelitis; (3) patients who cannot tolerate surgery due to severe organic diseases; and (4) patients who cannot undergo regular follow-up. After admission, we have performed a randomized division for included individuals to two different groups (routine group and 3D printing group). Specifically, 12 patients were enrolled into the routine group and 15 patients were enrolled into the 3D printing group, and further underwent the corresponding surgical methods, regardless of the severity of deformity. All patients included in this research have signed the informed consents, and the research was approved by Jiangxi Provincial People's Hospital Affiliated to Nanchang University.

### 2.2. PSI Osteotomy Guide Fabrication and Simulation Operation

**Figure 1:**
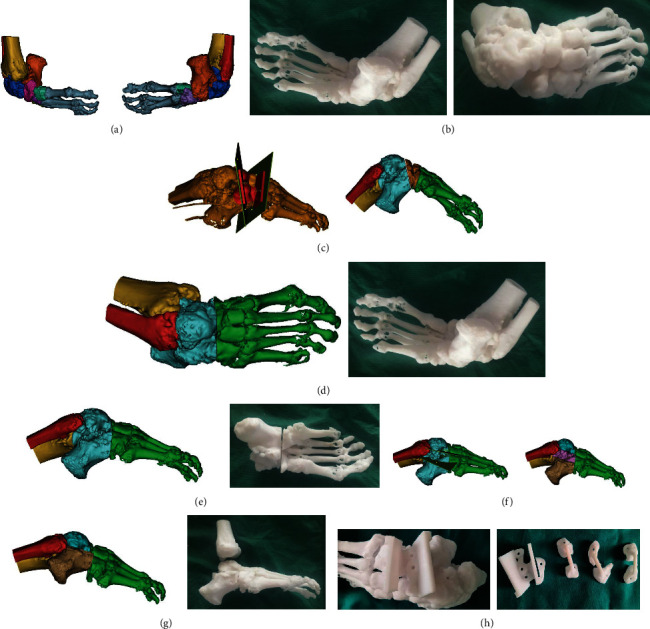
Design and fabrication processes of the 3D printing models and guides. (a) According to the imported CT data, the deformed structure of the ankle joint was simulated in Mimics19.0 software. (b) 1 : 1 reference model printed based on the simulation result. (c) In order to correct the deformity of arch elevation, the osteotomy planes were defined on the joint surfaces. (d) The simulated osteotomy for correcting the deformity of arch elevation was performed at the defined osteotomy planes, and the reduction was performed after osteotomy (frontal view). (e) The result of reduction after the simulated osteotomy (lateral view). (f) In order to correct the deformity of talipes varus, the osteotomy planes were defined on the joint surfaces. (g) The simulated osteotomy for correcting the deformity of talipes varus was performed at defined osteotomy planes, and the reduction was performed after osteotomy. (h) The osteotomy model and guides fabricated by the 3D printing technique.

All of the patients were routinely examined by CT scan and anteroposterior and lateral X-rays of malformed lower limb at the time of admission. The CT scanning data of 15 patients in the 3D printing group were gathered by dual-source 64-slice spiral CT system (Siemens, Munich, Germany). The exact scanning parameters of CT system included the voltage of 120 KV and the pitch of 0.625 mm. Then, the data were further imported into the Mimics 19.0 software (Materialise, Leuven, Belgium) in Digital Imaging and Communications in Medicine (DICOM) format by the professional orthopedic 3D medical engineers, so as to reconstruct the 3D model of malformed lower limb. On one hand, the osteotomy calculations were conducted by the professional orthopedic surgeons in our treatment team, and the specific observational indicators, such as the corrections of the deformities of talipes varus and arch elevation, were considered to achieve the desired corrections. On the other hand, with the close cooperation of orthopedic surgeons and orthopedic 3D medical engineers in our treatment team, the design thinking was conceived by the orthopedic surgeons and communicated with the 3D medical engineers face to face for practical operation, while the dedicated software operation was carried out by the 3D medical engineers. Furthermore, in order to correct the deformities of talipes varus and arch elevation caused by ATE malformation, the osteotomy planes were defined by the orthopedic surgeons at different joint surfaces, respectively, and the consistent shape of guides was established individually. Meanwhile, the data of Kirschner wire guide holes on each guide were also improved, respectively. Then, the further simulated osteotomy was performed, and the osteotomy surfaces of each joint were aligned to make the lower limb correct to the neutral position. After Boolean operation (through the operation of union, difference, and intersection of more than two objects, the new form of items can be obtained), the boundaries of guides were further trimmed, and all guide holes were penetrated through, thereby completing design and production of guides. Finally, the obtained data were saved in STereoLithography (STL) format and further imported to the 3D printer (Waston Med, Inc., Changzhou, Jiangsu, China) to print out the target models and the PSI osteotomy guides, with the materials of photosensitive resin (Figure 1).

### 2.3. Surgical Procedures

**Figure 2:**
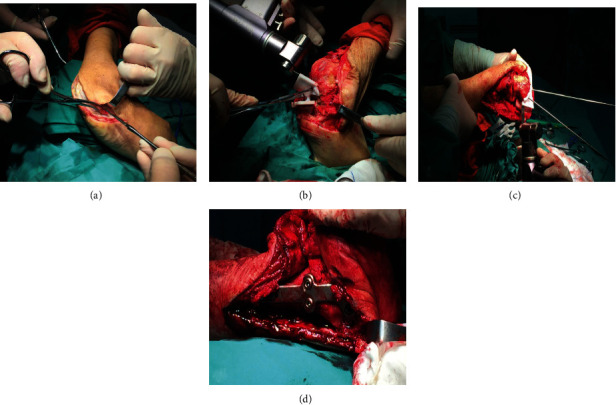
Intraoperative photographs of operation assisted by the 3D printing assisted PSI osteotomy guide. (a) The arc-shaped incision was made from the dorsolateral side of the foot to about 2 cm below the lateral malleolus. (b) and (c) The PSI osteotomy guide was fixed with the preset Kirschner wire guide holes on the binding surfaces, and the precise osteotomy was performed under the guidance of guides. (d) The reconstruction plate was used for the shaping and fixation of ATE malformation.

Senior surgeons in the same treatment group completed all the surgical procedures of all patients in this research. Under the general anesthesia or epidural, the patient was placed in the supine position, and the blood circulation of proximal thigh was blocked by the pneumatic tourniquet. Then, an arc-shaped incision was made from the dorsolateral side of the foot to about 2 cm below the lateral malleolus, and the flap was further freed to expose the tissues of ATE malformation. Then, according to the preoperative physical and imaging examinations, the degree of inversion and soft tissue contracture was measured, and the soft tissue release was performed, including the Achilles tendon lengthening, the extension of posterior tibial tendon, and the subcutaneous release of medial plantar, so as to maximize the muscle strength and obtain the balance. Regardless of the severity of deformity, the determination of osteotomy lines and the placement of PSI osteotomy guide were directly according to the outcomes of preoperative design and simulation in 3D printing group. Subsequently, two 2.0 mm Kirschner wires were then drilled into the model along with the guide hole to set each guide. After ensuring that the PSI osteotomy guide was matched and firmly fixed, the pendulum saw was used to carry out the precise osteotomy along with the PSI osteotomy guide (Figure 2). However, the determination of osteotomy lines was mainly based on preoperative planning and intraoperative attempts in the routine group, and the main procedures included correcting the deformities of talipes varus and arch elevation. After confirming the orthopedic osteotomy was satisfactory, the talocalcaneal and calcaneocuboid joints were fixed with hollow screws, and the talonavicular joint was fixed with door-shaped screw. Ultimately, the reconstruction plates (Dongya Med, Inc., Shenyang, Liaoning, China) were selectively used for the shaping and fixation according to the specific degree of deformities.

### 2.4. Postoperative Managements

There was no significant difference in postoperative managements between the two groups. After operation, the ankle joint of the affected side was fixed in 90° metatarsal flexion with lower leg plaster, and the affected limb was raised to observe the peripheral blood supply and skin sensation. Furthermore, the postoperative anterior and lateral X-rays and CT of the affected ankle joint were reexamined regularly. If the osteotomy end failed to heal at 9 months after the operation, it was regarded as the osteotomy end nonunion. Until the bony fusion was confirmed by the postoperative imaging examination, the plaster was demolished and the patients were instructed to begin partial rehabilitation training. In terms of the follow-up, patients in two groups were all regularly followed up within 1, 3, 6, 12, 24, and 36 months after the surgery. During follow-ups, the incision, skin healing, range of ankle joint motion, complications, and the time to obtain bony fusion were also regularly observed and recorded.

### 2.5. Parameter Assessment

The demographic data and ATE malformation characteristics between the two groups were recorded. Moreover, the parameters, including operative time, intraoperative blood loss, and range of ankle joint motion, were further observed and recorded. At the last follow-up, the functional outcomes of ATE malformation were evaluated by AOFAS [21] and ICFSG scoring systems [22]. In the ICFSG scoring system with a total score of 60, 0 is regarded as normal, 0-5 is regarded as excellent, 6-15 is regarded as good, 16-30 is regarded as fair, and >30 is regarded as poor.

### 2.6. Statistical Analysis

Data presented in this current research were statistically analyzed by the SPSS 24.0 software (SPSS, Inc., Chicago, USA) and exhibited as mean ± standard deviation (SD) or count (percentage). The statistical methods of Chi-squared test, Fisher exact test, and Student's *t* test were applied in this research. The independent *t* test was used to assess the continuous variables, and the chi-square test or Fisher exact test was used to assess the categorical variables of different parameters collected between the two groups. *P* value < 0.05 was represented as statistically significant.

## 3. Results

### 3.1. Demographic Data and ATE Malformation Characteristics

Table 1 presents the demographic data and ATE malformation characteristics of two groups in this research. However, there was no significant difference between the two groups in age, gender, ATE malformation side, and causes of ATE malformation (*P* > 0.05 for all).

### 3.2. Clinical Data and Functional Outcomes

Clinical data and functional outcomes of the two groups are summarized in Table 2. In terms of operative time, (96.3 ± 14.2 min) in the 3D printing group was significantly less than (122.9 ± 18.3 min) in the routine group (*P* < 0.001). In terms of intraoperative blood loss, the difference between the 3D printing group (98.6 ± 18.7 ml) and the routine group (126.5 ± 23.2 ml) was statistically significant (*P* < 0.001). However, there was no significant difference in follow-up time, time to obtain bony fusion, range of ankle joint motion, and AOFAS score at last follow-up (*P* all > 0.05). As for the ICFSG score at last follow-up, 5 patients were evaluated as excellent, 9 patients were good, and 1 patient was fair in the 3D printing group. In the routine group, 3 patients were evaluated as excellent, 6 patients were good, and 3 patients were fair. The rate of excellent and good outcomes of the 3D printing group was 93.3%, which was higher than that of the routine group (75%, *P* = 0.019). In addition, types and number of operations performed in each group were exhibited in supplementary table (available here).

### 3.3. Complications

As shown in Table 3, the total rate of complications of the 3D printing group and routine group was 13.3% (2/15) and 16.7% (2/12), respectively, and there was no significant difference (*P* = 0.291).

### 3.4. Typical Case

**Figure 3:**
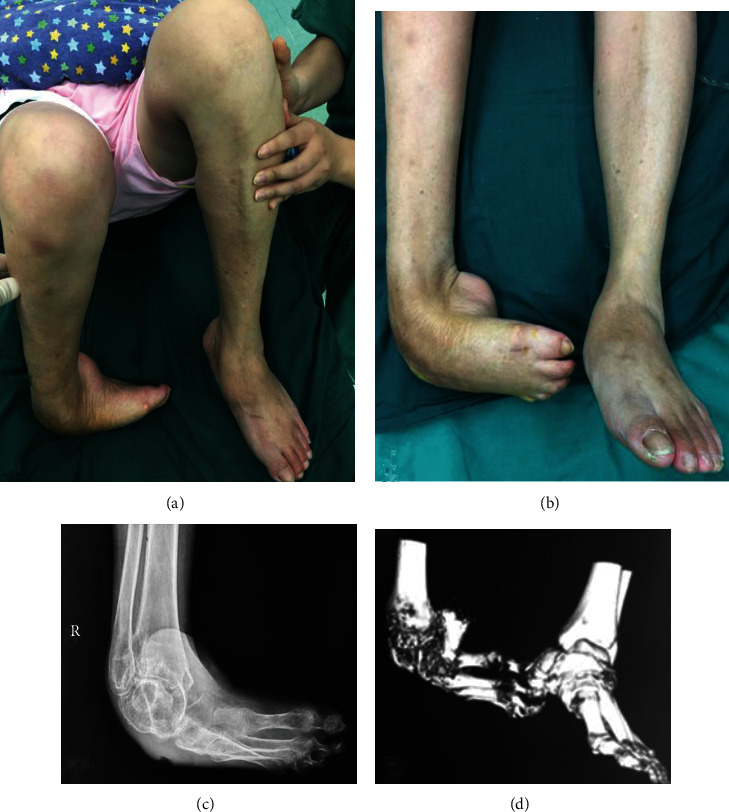
Female, 57 years old, who has suffered from CTE in right side for 50 years. (a) The patient has not paid enough attention to the CTE and walked on the back of foot for 20 years. (b) Preoperative appearance, and the physical examination revealed that the right ankle joint was stiff, the subtalar joint had no range of motion, and the knee tendon reflex was hyperactive. (c) and (d) Preoperative X-ray and 3D reconstruction CT scanning of right ankle joint indicated the severe ATE malformation of the patient.

**Figure 4:**
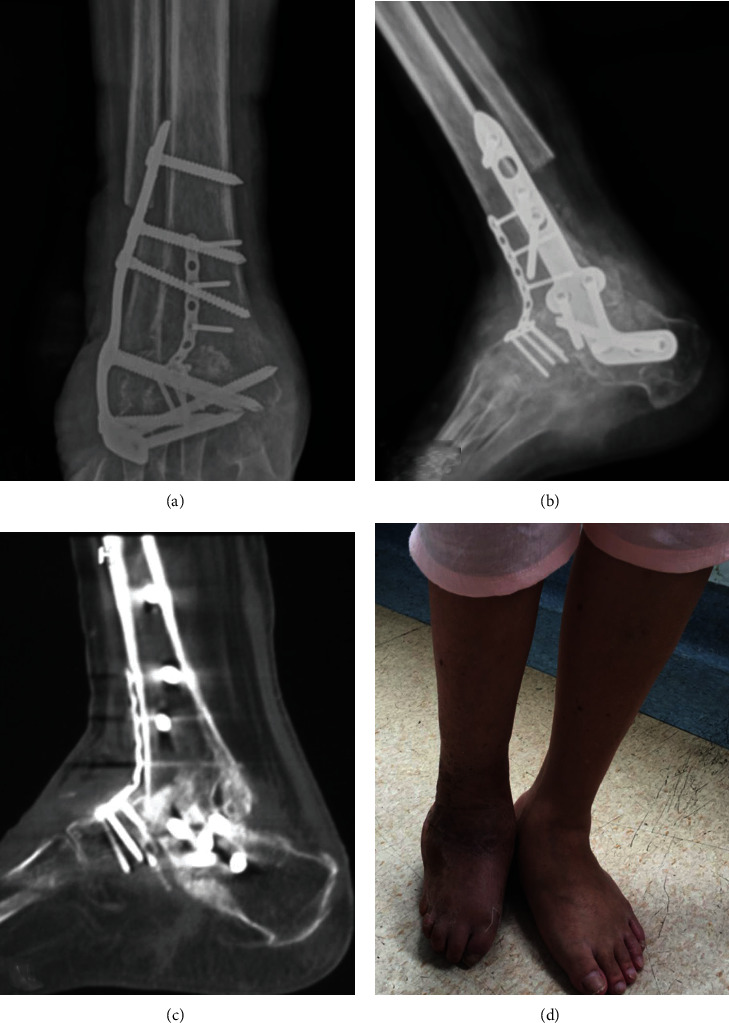
Postoperative imaging examinations and general appearance of right ankle joint. (a) and (b) Postoperative X-rays indicated that the osteotomy end was well aligned and the fixation effect was reliable. (c) At 12 weeks postoperatively, the osteotomy end obtained the bony fusion confirmed by CT scanning. (d) The general appearance of the right ankle joint at the last follow-up.

Female, 57 years old, who has suffered from congenital talipes equinovarus (CTE) in right side for 50 years. The patient has not paid enough attention to it and walked on the back of foot for 20 years. Later, the patient was admitted to our hospital for more than half a year due to the right foot pain and limited activity (Figure 3). Physical examination revealed that the right ankle joint was stiff, the subtalar joint had no range of motion, and the knee tendon reflex was hyperactive. After the treatment of osteotomy assisted by PSI osteotomy guide, further tibiotalocalcaneal arthrodesis was then performed, and the postoperative imaging examination indicated that the osteotomy end was well aligned and the fixation effect was reliable. At the follow-up of 12 weeks, the osteotomy end obtained the bony fusion (Figure 4). At the last follow-up, the AOFAS score was evaluated as 81 points, and the ICFSG score was 12 points, which was evaluated as good level.

## 4. Discussion

To sum up, this study demonstrated the safety, accuracy, and reliability of 3D printing assisted PSI osteotomy guide for correcting the ATE malformation. Compared with the routine group, the 3D printing group exhibits the superiorities of shorter operative time, less intraoperative blood loss, higher rate of excellent, and good outcomes expressed by the ICFSG score at last follow-up. Moreover, the operation assisted by the PSI osteotomy guide for correcting the ATE malformation is novel and feasible, which might become an effective method to polish up the precise osteotomy of ATE malformation and polish up the clinical efficacy.

In this current research, we compared the clinical efficacy and prognosis of routine ATE deformity correction surgeries and operation assisted by PSI osteotomy guide for the treatment of ATE malformation patients. In the 3D printing group, the personalized PSI osteotomy guide fabricated by 3D printing technique can be closely fitted to each joint surface. In all 15 patients, the osteotomy was performed successfully in one time, avoiding the repeated adjustments and tests during operation, which contributes to a more standardized and simpler operation. Meanwhile, it also reasonably explained that the parameters of operative time and intraoperative blood loss in the 3D printing group were less than that in the routine group (*P* < 0.001). In addition, the PSI osteotomy guide can be fixed with the preset Kirschner wire guide holes on the binding surfaces, which can effectively prevent the pendulum saw from slipping during the process of operation and producing deviation. Hence, after the processes of rigorous design and standardized operation, the accurate osteotomy of ATE malformation can be realized, and the clinical efficacy was equivalent to the preoperative planning. Meanwhile, the 3D printing group also exhibited a higher rate of excellent and good outcomes than the routine group in ICFSG score at last follow-up (93.3% versus 75.0%, *P* = 0.019).

In addition, although the 3D printing assisted PSI osteotomy guide has been diffusely applied in several clinical studies, such as the cubitus varus deformity, developmental dysplasia of the hip (DDH), spinal scoliosis, hallux valgus, and other deformities [20, 23–25], while its application in the correction of ATE malformation has been rarely reported. Previously, Windisch et al. [26] applied 3D printing technique to fabricate a physical model of ATE malformation with a magnification of 4 times for surgeons to accurately analyze all bone and joint deformities and perform preoperative planning, but this application only played the most basic role of 3D printing technique. Moreover, Gozar et al. [16] and Barker et al. [27] applied computer modeling analysis technique to the correction of TE malformation and obtained the satisfactory short-term outcomes, but still lacked a long-term prognostic comparison with the routine group. In this research, there was no significant difference between the two groups in total rate of complications (13.3% vs. 16.7%, *P* = 0.291). This outcome was consistent with the research results of Zhang et al. [28] used the 3D printing technique for the patients with complicated ankle fractures, indicating that the 3D printing technique presents no apparent superiorities in avoiding complications. Furthermore, at the last follow-up, there was no significant difference in the range of ankle joint motion, including the dorsal expansion, plantarflexion, inversion, and eversion, between the two groups (*P* all > 0.05). This may be attributed to the similar time of obtaining bony fusion and taking the similar programs of rehabilitation training between the two groups, and both groups presented relatively excellent postoperative functional outcomes. In addition, it is worth noting that the mismatch between the ICFSG and AOFAS scores in the functional outcome evaluation is not correlated to difference in range of ankle joint motion, including the dorsal expansion, plantarflexion, inversion, and eversion. This outcome might be relevant to the difference in the time of demolishing the plaster and beginning the rehabilitation training.

Ultimately, it is essential to further point out and recognize the drawbacks of this research. For one thing, the mean follow-up time of this research was about 2 years. At the last follow-up, the prognosis of partial patients with ATE malformation (3 patients in routine group and 1 patient in 3D printing group) was evaluated as the fair, and it is essential to conduct further follow-up observation for a longer time in future. For another thing, this current research is the retrospective study with relatively small sample size. In order to further verify the clinical efficacy and prognosis of 3D printing assisted PSI osteotomy guide for the correction of ATE malformation, multicenter prospective studies and larger sample size data are still needed. Moreover, the specific comparison of pre- and postoperative radiological lower limb alignment parameters between the two study groups is also of great significance and interesting, which is expected to be further improved in future researches.

## 5. Conclusions

Clinical application of 3D printing assisted PSI osteotomy guide for correcting the ATE malformation is safe, precise, and dependable. The 3D printing group exhibits the superiorities of shorter operative time, less intraoperative blood loss, and higher rate of excellent and good outcomes presented by ICFSG score at last follow-up than the routine group. The operation assisted by 3D printing PSI osteotomy guide for correcting ATE malformation is novel and feasible, which might be an effective method to polish up the precise osteotomy of ATE malformation and enhance the clinical efficacy.

## Figures and Tables

**Table 1:** Comparisons of demographic data and ATE malformation characteristics between two groups.

Characteristics	Routine group (*n* = 12)	3D printing group (*n* = 15)	*P* value
Mean age (range), years	54.7 ± 12.3 (45-68)	56.1 ± 13.2 (43-69)	0.579
Gender, *n* (%)			0.743
Male	5 (41.7)	7 (46.7)	
Female	7 (58.3)	8 (53.3)	
ATE malformation side, *n* (%)			0.285
Left	8 (66.7)	9 (60.0)	
Right	4 (33.3)	6 (40.0)	
Causes of ATE malformation, *n* (%)			0.641
Neglected treatment of CTE	12 (100)	15 (100)	
Recurrence of CTE after treatment	0	0	
Sequelae of polio	0	0	
Trauma	0	0	

Note: CTE: congenital talipes equinovarus; ATE: adult talipes equinovarus.

**Table 2:** Comparison of clinical data and functional outcomes between two groups of ATE malformation.

	Routine group (*n* = 12)	3D printing group (*n* = 15)	*P* value
Operative time, min	122.9 ± 18.3	96.3 ± 14.2	<0.001
Intraoperative blood loss, ml	126.5 ± 23.2	98.6 ± 18.7	<0.001
Follow-up time, month	26.1 ± 7.6	25.3 ± 6.9	0.352
Time to obtain bony fusion, week	13.3 ± 3.1	12.6 ± 2.7	0.243
Range of ankle joint motion at last follow-up °			
Dorsal expansion	23.6 ± 3.4	24.2 ± 3.8	0.371
Plantarflexion	26.8 ± 3.7	27.4 ± 2.9	0.253
Inversion	24.3 ± 3.1	25.5 ± 3.5	0.162
Eversion	27.3 ± 3.4	28.1 ± 3.2	0.527
AOFAS score at last follow-up, point	77.8 ± 9.1	78.5 ± 8.5	0.136
ICFSG score at last follow-up, *n* (%)			
Excellent	3 (25.0)	5 (33.3)	0.257
Good	6 (50.0)	9 (60.0)	0.632
Fair	3 (25.0)	1 (6.7)	0.876^a^
Poor	0	0	—
Rate of excellent and good outcomes, %	75.0	93.3	0.019

^a^ *P* value for continuity-corrected chi-squared test. Note: ICFSG: International Congenital Clubfoot Study Group; AOFAS: American Orthopedic Foot and Ankle Society; ATE: adult talipes equinovarus.

**Table 3:** Comparison of complications between two groups of ATE malformation.

Complications	Routine group (*n* = 12)	3D printing group (*n* = 15)	*P* value
Superficial infection	0	1 (6.7)	1.000^a^
Deep infection	1 (8.3)	0	1.000^a^
Skin necrosis	0	0	—
Nerve injury	0	0	—
Vascular injury	0	0	—
Osteotomy end nonunion	0	0	—
Anklebone stiffness	1 (8.3)	1(6.7)	1.000^a^
Total	2 (16.7)	2 (13.3)	0.291

Values are expressed as *n* (%); ^a^*P* value for Fisher's exact test. Note: ATE: adult talipes equinovarus.

## Data Availability

The data used to support the findings of this study are available from the corresponding author upon request.

## Supplementary Materials

Supplementary Materials Supplementary table: the types and number of operations performed in each group.
