# Supplementary material for: The Novel Application of Three-Dimensional Printing Assisted Patient-Specific Instrument Osteotomy Guide in the Precise Osteotomy of Adult Talipes Equinovarus
Source: Biomed Res Int. 2021 Dec 2;2021:1004849. doi: 10.1155/2021/1004849 (PMC8660203; doi:10.1155/2021/1004849)
Supplement: Supplementary Materials — Supplementary table: the types and number of operations performed in each group. [file 1004849.f1.docx]

**Supplementary table: The types and number of operations performed in each group**

|  | Routine group (*n*=12) | 3D printing group (*n*=15) |
| --- | --- | --- |
| Type, *n* |  |  |
| Tibiotalocalcaneal arthrodesis | 3 | 2 |
| Achilles tendon lengthening+Tibiotalocalcaneal arthrodesis | 2 | 1 |
| Balance the muscle force of internal and external inversion+Tibiotalocalcaneal arthrodesis | 0 | 0 |
| Achilles tendon lengthening+Balance the muscle force of internal and external inversion+Tibiotalocalcaneal arthrodesis | 2 | 3 |
| Triple arthrodesis | 1 | 2 |
| Achilles tendon lengthening+Triple arthrodesis | 1 | 2 |
| Balance the muscle force of internal and external inversion+Triple arthrodesis | 0 | 0 |
| Achilles tendon lengthening+Balance the muscle force of internal and external inversion+Triple arthrodesis | 3 | 5 |
